# Bone cement-enhanced sternal closure technique in cardiac surgery: effects on sternal union, pain and life quality

**DOI:** 10.1186/1749-8090-8-182

**Published:** 2013-08-07

**Authors:** Zehra Bayramoglu, Yasemen Durak, Muhammed Bayram, Onur Levent Ulusoy, Barıs Caynak, Ertan Sagbas, Belhan Akpınar

**Affiliations:** 1 Department of Cardiovascular Surgery, Florence Nightingale Hospital, Abide-i Hurriyet Street No:164 Sisli 34381, Istanbul, Turkey; 2 Department of Radiology, Florence Nightingale Hospital, Istanbul, Turkey

## Abstract

**Background:**

Median sternotomy provides excellent access to all mediastinal structures in patients undergoing conventional cardiovascular surgery. Although this incision technique is associated with relatively lower complication rates, certain complications such as the sternal dehiscence may pose serious health consequences. In this regard, considerable effort has been paid to develop techniques aiming to improve sternal healing and to enhance postoperative recovery after conventional cardiac surgery. Among these, kryptonite bone cement, a biocompatible polymer with improved mechanical properties when combined with a standard wire cerclage, represents a promising novel approach that may help prevent sternal dehiscence. In this study, the effects of this particular type of bone cement on sternal healing, postoperative pain, and quality of life have been evaluated.

**Methods:**

Kryptonite bone cement enhanced sternal closure was employed in a total of 100 patients undergoing conventional cardiac surgery between November 2009 and June 2012. Of these patients, 50 expressed their willingness to participate in this study. Each participant underwent a computerized tomography imaging for the radiological assessment of sternal healing. Pain and life quality of these patients have been evaluated by Wong-Baker faces pain scale and SF-36 health survey questionnaire, respectively.

**Results:**

Mean duration of follow-up was 20.14 ± 7.36 months (range: 10–32). Mean age and body mass index were 71.32 ± 7.23 years (range: 55–85) and 28.34 ± 2.62 (21–34) kg/m^2^, respectively. Elderly patients (≥70), females and those with chronic obstructive pulmonary disease (COPD) comprised 64%, 26% and 40% of the study population, respectively. No patients had findings suggestive of dehiscence on CT images. No patients reported severe pain (i.e. all patients had a Wong-Baker faces pain scale score <4). Elderly (≥ 70 yr) subjects had better quality of life scores as compared to the remaining group of patients (< 70 yr) according to SF-36 Health Survey results. Vitality and emotional role scores were lower (63.5 ± 25.5, p = 0.018 and 41.7 ± 23.3, p = 0.001, respectively) in female patients. Patients with COPD had lower quality of life scores than those without COPD, particularly with respect to general health scores (73.3 ± 18.5; p = 0.012).

**Conclusions:**

Kryptonite bone cement, when combined with a standard wire cerclage, enhances mechanical strength, prevents sternal dehiscence, reduces postoperative pain and improves quality of life after conventional cardiac surgery. Long-term studies are warranted to better define the role of kryptonite bone cement in the prevention of sternal dehiscence.

## Background

Median sternotomy for cardiac surgery is a frequently used incision technique by cardiovascular surgeons owing to several advantages it offers such as the shortened duration of opening and closure, as well as excellent exposure [1]. Every year more than 750.00 median sternotomies are performed in the United States. The reported figures for sternal dehiscence among these patients vary between 0.5% and 8%, with a mortality rate between 10 and 40% [2,3]. This complication often is observed in the first two postoperative weeks and is more likely to occur in those who are over 75 years of age, or have morbid obesity, a BMI ≥ 30 kg/m^2^, or a history of osteoporosis.

The single most important factor in preventing sternal dehiscence and mediastinitis is achieving a stable sternal approximation, and the traditional sternal closure techniques may not always provide optimal fixation.

The objective of our study was to evaluate the benefits of a particular sternal closure technique on sternal healing, postoperative pain and quality of life in patients undergoing cardiac surgery. Essentially, this technique is based on enhancement of the standard wire cerclage through the use of a special bone cement, which is a biocompatible polymer composed of naturally occurring fatty acids and calcium carbonate, derived from castor oil (Kryptonite, Doctors Research Group Inc, Southbury, CT).

## Methods

Sternal closure enhanced by kryptonite bone cement was implemented in a total of 100 patients undergoing conventional cardiac surgery between November 2009 and June 2012. This study was approved by ethical boards of Bilim University Hospital and each pateints provided written informed consent. Computed tomography, Wong-Baker faces pain scale, and SF-36 health survey questionnaire have been utilized to assess the benefits of this technique on sternal union, postoperative pain and quality of life. Following discharge, a telephone call invitation was made for study participation to every patient, of whom 50 accepted to participate and give written consent for the study. Twelve patients could not be contacted due to changes in contact information. The remaining declined participation mostly due to radiation concerns associated with the use of computed tomography or due to their remote settlement from the hospital.

Five patients between 80 and 89 years of age underwent a vertebral operation during the early postoperative period, on average 1 week after the conventional cardiac surgery. Sternal dehiscence did not occur in these patients and three of them consented to participate in the study.

A high-risk patient had sternal dehiscence secondary to mediastinitis and died due to heart failure during early postoperative period. Superficial wound infections were observed in two patients with diabetes mellitus, but these patients were among those who had initially refused to participate in the study.

Re-exploration surgery was performed in two patients due to bleeding. These two cases were excluded from the study because the integrity of kryptonite bone cement was disrupted and a second application could not be performed due to personal financial constraints.

### Data collection

Written consent for the use of bone cement was obtained from all 100 patients before surgery. However, since study participation required further procedures, additional written consent for the study was obtained from 50 patients who showed their willingness for participation. Hospital database was used to collect information on preoperative, intraoperative and postoperative characteristics of the patients. Follow up visits were performed in office conditions. Sternal union was ascertained with physical examination and computed tomography. Pain and quality of life scores were assessed using Wong-Baker faces pain scale and SF-36 health survey questionnaire, respectively.

### Inclusion/exclusion criteria

First-time or redo patients between 50 and 90 years of age presenting with an indication for on-pump cardiac surgery to be carried out through median sternotomy who provided informed consent to participate were included in the study. In addition, each patient should have at least one of the following criteria: age ≥75 years, body mass index (BMI) ≥30 kg/m^2^, osteoporosis, limited mobility to plan a vertebral surgery in early postoperative period, active smoking until time of hospitilization, chronic obstructive pulmonary disease. Patients with postoperative re-exploration for bleeding were excluded.

### Surgical technique

Kryptonite bone cement (Doctors Research Group Inc, Southbury, CT) is a biocompatible polymer composed of naturally occurring fatty acids and calcium carbonate, derived from castor oil. The porous network within the product allows osteointegration with host bone over time, without fibrosis or inflammation.

**Figure 1:**
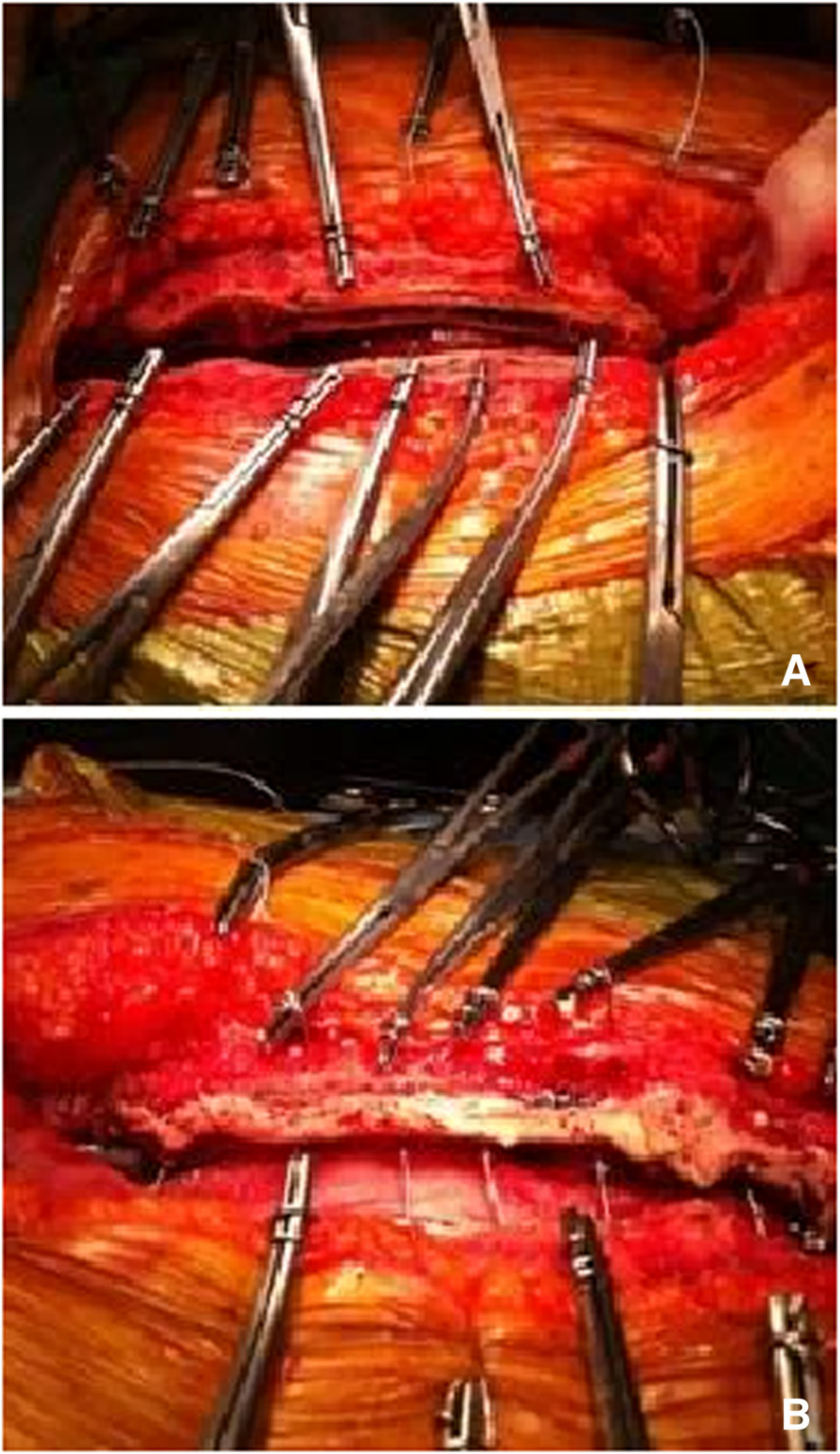
Severely osteoporotic sternum (A); application of Kryptonite bone cement (B).

Following standard cardiopulmonary bypass, sternal closure was performed in all patients using 7-wire interrupted cerclage with (No:6) 18-gauge stainless steel. Then, trabecular bone was cleansed with a sterile brush and irrigated with saline solution to expose the bone trabecular interface. The Kryptonite bone adhesive was mixed for 5 minutes, and then 5 ml of it was applied as a thin layer to each hemisternum. It is an injectable liquid for up to 8 minutes after mixing three components, then, transitions into a highly adhesive taffy state 8–15 minutes after mixing. Through polymerization, it attains bone-like rigidity and strength (Figure 1). The sternum was closed before the cement completely set. Excess cement under the sternum was removed by a gauze prior to closing. Similarly, excess cement on the sternum was removed by a wet gauze. The material was used as a bone filler, as well as for bleeding control. Other hemostatic agents including bone wax were not used. Importantly, the adhesive can be easily divided with a sternal saw to facilitate rapid chest reentry if re-exploration is needed.

Fifty patients underwent computed tomography for the evaluation of sternal healing. SF 36 Health Survey and Wong-Baker faces tests were used for evaluation of pain and life quality, respectively, after bone cement enhanced sternum closure.

### Multislice computed tomography (MCT)

CT offers significant advantages in the evaluation of suspected sternal disease and provides superior image quality compared to plain radiography [4]. In this study, a 16-slice CT device (Somatom Senstation 16, Siemens Medical Solutions) was used for thoracic imaging at follow-up examinations. Retrospective multiplanar reconstructions were obtained using a lung algorithm with slices of 1 mm cross-thickness and 1 mm spacing. Reformatted axial images were constructed with 1 mm spacing.

CT scans were reviewed by the same radiologist for the presence of retained bone cement, bone healing, replacement of cement with bone over time, evidence of osteomyelitis, nonunion of the sternum, and new bone density. Sternal gap was defined as a gap between the sternal halves visible on CT. Dehiscence was defined as clinically identifiable disruption of the stable fixation on the sternum. A definitive diagnosis for dehiscence was made by clinical evidence in combination with the CT findings.

### Quality of life and pain

Pain perception and the perceived quality of life are subjective measures that exhibit significant inter-personal variability. For instance, a subject with a serious health condition may report a relatively good quality of life, while another with a minor disability may be found to experience a poor quality of life. Short form 36 (SF-36) is a widely used tool in clinical studies to assess the quality of life and general health perception. It can be used to assess the physical and mental health as well as how the patient perceives his or her health or illness [5]. This questionnaire is also commonly used after cardiovascular surgery to evaluate the life quality of a patient [6].

SF-36 consists of 36 questions in 8 domains. These 8 domains include physical functioning (being able to perform physical activities), physical role (being able to do his/her job and daily activities), bodily pain (no pain or limitations due to pain), general health perception (evaluates personal health), vitality (enjoying life), social functioning (performing normal social activities or not), emotional role (problems with work or daily activities as a result of emotional problems), and mental health (feeling happy, peaceful and calm). Three domains (i.e., physical functioning, physical role and bodily pain) show high correlation with the physical component (PCS). Mental health and emotional role items mostly correlate with the mental component (MCS). Vitality, general health, and social functioning correlate with both. Scores are converted to a 0–100 scale, which allows numerical assessment of the domains. Higher scores indicate less limitation and disability.

Pain and discomfort were measured by Wong-Baker faces pain scale is a self-rated tool, which utilizes a number of face depictions that range from happy to tearful (0–10). Reliability of the scale has been demonstrated in a well-designed study [7]. It is a widely used pain measurement tool due to its established validity and reliability in different patient groups.

### Statistical analysis

All analyses were performed using SPSS (Statistical Packages for the Social Sciences) for windows, version 17.0. Mann Whitney U test was used to compare the demographic and clinical characteristics of participants. Chi-Square test was used for the comparisons of frequencies between the groups. All data are expressed as mean ± standard error of the mean. *P* values less than 0.05 are considered statistically significant.

## Results

Demographic characteristics of the 50 participants are summarized in Table 1. Mean age was 71.32 ± 7.23 years (range, 55–85) and mean body mass index was 28.34 ± 2.62 kg/m^2^ (range, 21–34). More than 50% of the patients were overweight or obese. Thirty-six percent of the patients had diabetes and three patients had arthritic conditions including rheumatoid arthritis and gout.

**Table 1:** Demographic properties of the patients

**Preoperative characteristics**	**N(50)**	**%**	**Mean**
**Age**			71.32 ± 7.23(55–85)
<70	18	36	
<70	32	64	
**Sex**			
Male	37	74	
Female	13	26	
**Body mass index (kg/m**^**2**^**)**			28.34 ± 2.62(21–34)
Normal Weight	5	10	
Overweight	32	64	
Obesity	13	26	
**Diabetus mellitus**	18	36	
**Hypertension**	32	64	
**Chronic obstructive pulmonary disease**	20	40	
**Arthritis**	3	6	
**Cerebrovascular disease**	2	4	
**Chronic renal failure**	0	0	
**Acetylsalicylic acid therapy**	36	72	
**Heparin therapy**	13	26	

Mean duration of follow-up was 20.14 ± 7.36 months (range: 10–32). Two patients were redo cases and underwent valvular and aortic procedures. In 64% of the subjects, a coronary artery bypass surgery was performed, with bilateral use of internal mammary arteries in five cases. The mean volume of postoperative bleeding in the intensive care unit was 185 ± 69 ml (range, 100–400). Seventy-two percent of the patients were on aspirin and 26% were on heparin preoperatively. None of the participants required postoperative re-exploration for bleeding. Intra- and post-operative characteristics of the patients can be seen in Table 2.

**Table 2:** Intraoperative and postoperative patient data

**Variable**	**N(50)**	**%**	**Mean**	**SD**	**Min.**	**Max.**
**Surgery**						
Coronary artery surgery	32	64				
Valve surgery	5	10				
Aortic surgery	1	2				
CABG plus valve surgery	9	18				
Myxoma	1	2				
CABG plus aortic surgery	2	4				
**Cardiopulmonary bypass time (minutes)**			72.05 ±	34.35	(30 -	185)
**AC time (minutes)**			48.15 ±	25.44	(12 -	125)
**Ejection fraction (%)**			50.85 ±	7.88	(32 -	65)
<30	0	0				
30-50	26	52				
>50	24	48				
**Postoperative drainage (ml)**			185 ±69		(100 -	400)
**Redo cardiac surgery**	2	4				
**ICU stay (hours)**			29.26 ± 8.8			
**Entubation time (hours)**			4.42 ± 1.73			
**Hospital stay (day)**			6.12 ± 1.43			
**Clinical examination of sternum**						
Minimally unstable	2	4				
**CT analysis**						
Sternal union	47	94				
Gaps	2	4				
Dehiscence	0	0				
**Follow-up time (months)**	-	-	20.14 ±	7.36	(10 -	32)

**Figure 2:**
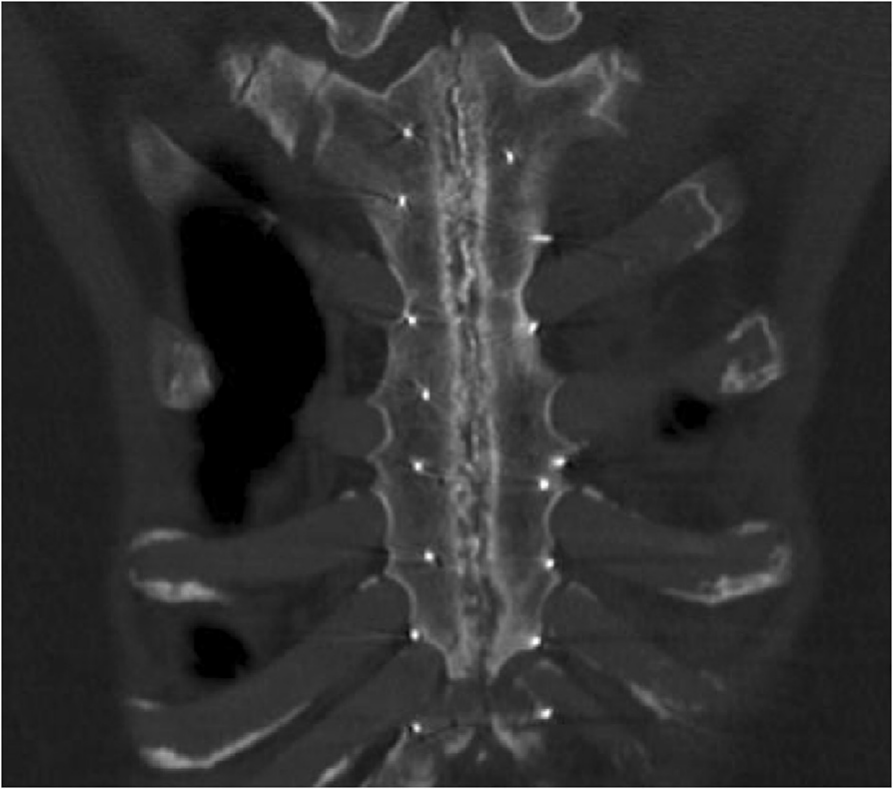
Computed tomography of chest showing sternal union in a patient who had adhesive-enhanced closure.

MCT scans showed the signs of retained kryptonite cement, bone healing, and increased radiodensity between the sternal edges, suggestive of new bone formation. Physical examination showed minimal sternal instability in two patients that correlated with the gaps between the sternal halves seen on CT. However, no patients had dehiscence (Figure 2).

Percentage of patients who reported no hurt (0), hurt little (2) and hurt little more (4) according to Wong-Baker faces pain scale was 80%, 16% and 4%, respectively. There was no patient who reported severe pain (4–10). Assessment of the association between risk factors and pain scores showed a significant correlation between the severity of pain and arthritis only (x^2^ = 9.18. p = 0.010). The three patients with arthritic conditions in our study population reported mild to moderate pain.

Life quality was evaluated by SF-36 Health Survey. In general, higher scores were obtained for physical and mental components, and the highest score was recorded for bodily pain. These findings show that two thirds of the patients were free from the pain after sternal closure with cement (79.24 ± 24.85. p = 0.042). Lowest scores were found for the emotional role (64.91 ± 45), probably associated with the predominance of frail elderly subjects among study participants (Table 3).

**Table 3:** **Evaluation of life quality (SF-36 Health Survey; 0–100**) **according to pain scale (Wong-Baker FACES pain rating scale; 0–10**)

	**No hurt (0)**		**Hurt little bit and little more (2–4)**		**Significance**	
	**(n = 38)**		**(n = 12)**			
**SF-36 scales**	**Mean**	**SD**	**Mean**	**SD**	**Z**	**p**
**Physical component**						
Physical functioning (PF)	87.37	20.3	75.95	28.09	−1.144	0.252
Role-Physical (RP)	73.68	45.24	76.19	43.64	−0.181	0.857
Bodily pain (BP)	92.74	12.91	79.24	24.85	**−2.031**	0.042*
General health (GH)	85.11	14.03	78.76	18.97	−0.901	0.368
**Mental component**						
Vitality (VT)	84.74	19.11	74.29	24.05	−1.414	0.157
Social functioning (SF)	81.58	22.96	71.43	33.34	−0.661	0.509
Role-emotional (RE)	64.91	45.1	63.49	45.83	−0.092	0.927
Mental health (MH)	79.58	15.94	76.76	15.32	−0.696	0.486

* = p < 0,05; **Z =** Mann–Whitney U Test.

The quality of life did not differ significantly between those aged 70 or over and those below 70, suggesting that kryptonite enhanced sternal closure is able to provide sternal stability and allows quick return to daily activities even in osteoporotic elderly patients (Table 4).

**Table 4:** **Comparison of life quality according to age (older or younger than 70**) **by SF-36 Health Survey**

	**<70 year**		**≥70 year**		**Significance**	
	**(n = 18**)		**(n = 32)**			
**SF-36 scales**	**Mean**	**SD**	**Mean**	**SD**	**z**	**P**
**Physical component**						
Physical functioning (PF)	80.56	23.00	82.05	27.15	−0.397	0.691
Role-physical (RP)	83.33	38.35	68.18	47.67	−1.087	0.277
Bodily pain (BP)	89.11	18.90	82.82	22.57	−1.131	0.258
General health (GH)	81.06	16.20	82.36	17.81	−0.425	0.671
**Mental component**						
Vitality (VT)	76.11	23.86	81.82	20.96	−0.951	0.342
Social functioning (SF)	75.69	30.76	76.70	28.16	−0.144	0.885
Role-emotional (RE)	66.66	43.65	61.11	47.49	−0.398	0.691
Mental health (MH)	77.78	16.07	78.36	15.35	−0.151	0.880

p > 0,05; **Z** = Mann–Whitney U Test.

Female patients had lower vitality (63.5 ± 25.5; p = 0.018) and emotional role (41.7 ± 23.3; p = 0.001) scores as compared to male subjects. The observed difference between sexes in these two domains representing the mental component of SF36 may be related to differential psychological reactions of the two sexes to surgery (Table 5).

**Table 5:** Comparison of life quality according to gender by SF-36 Health Survey

	**Male (n = 37)**		**Female (n = 13)**			
**SF-36 scales**	**Mean**	**SD**	**Mean**	**SD**	**z**	**p**
**Physical component**						
Physical functioning (PF)	83.67	25.90	74.50	22.17	−1.548	0.122
Role-physical (RP)	80.00	40.68	60.00	51.64	−1.249	0.212
Bodily pain (BP)	84.60	22.71	88.80	15.18	−0.188	0.851
General health (GH)	83.57	16.76	76.40	17.02	−1.133	0.257
**Mental component**						
Vitality (VT)	84.50	18.63	63.50	25.50	**−2.374**	0.018*
Social functioning (SF)	80.00	26.18	65.00	35.26	−1.127	0.260
Role-emotional (RE)	77.78	37.48	41.72	23.33	**−3.219**	0.001**
Mental health (MH)	80.13	14.95	72.00	16.22	−1.464	0.143

* = p < 0,05; ** = p < 0,01.

**Z** = Mann–Whitney U Test.

Quality of life in those subjects with chronic obstructive pulmonary disease (COPD) was lower as compared to those without COPD, particularly in terms of general health (73.3 ± 18.5; p = 0.012), which is physical domain of SF36. Even though all patients had Kryptonite bone cement, COPD had negative effect on life quality (Table 6).

**Table 6:** **Comparison of life quality according to the presence of COPD (Chronic obstructive pulmonary disease**) **by SF-36 Heath Survey**

	**COPD (−) (n = 30)**		**COPD (+) (n = 20)**			
**SF-36 scales**	**Mean**	**SD**	**Mean**	**SD**	**z**	**p**
**Physical component**						
Physical functioning (PF)	86.46	22.77	73.75	27.11	1.685	0.092
Role-physical (RP)	79.17	41.49	68.75	47.87	0.736	0.462
Bodily pain (BP)	88.17	22.22	81.88	19.04	1.798	0.072
General health (GH)	87.42	13.30	73.31	18.56	**2.504**	0.012*
**Mental component**						
Vitality (VT)	84.58	19.50	71.25	24.19	1.749	0.080
Social functioning (SF)	77.08	27.75	75.00	31.62	0.029	0.977
Role-emotional (RE)	66.66	45.05	60.42	45.90	0.606	0.544
Mental health (MH)	80.00	16.13	75.25	14.48	1.099	0.272

* = p < 0,05; **Z** = Mann–Whitney U Test.

## Discussion

Median sternotomy is a practical and quickly implemented incision technique that provides excellent access to all mediastinal structures and it is well tolerated by most patients undergoing conventional cardiovascular surgery. Although it is associated with relatively low complication rate, sternal dehiscence represents a potentially fatal serious condition [8]. Associated risk factors for sternal dehiscence are many and include osteoporosis, obesity, COPD, diabetes, corticosteroid use, off-midline sternotomy, poor nutritional status, prolonged surgery, persistent postoperative coughing, inappropriate timing of prophylactic antibiotic administration, septic operations, and many others [9]. Several of these risk factors are commonly coexistent in cardiac surgery populations.

A considerable effort has been paid to develop techniques aiming to improve sternal closure and to enhance postoperative recovery after conventional cardiac surgery [10]. In this regard, kryptonite represents a promising innovative bone cement material, which is biocompatible, easy to mix and deliver, and adds very little time to the total duration of surgery. Used in conjunction with conventional sternal wires, it is applied within the trabecular compartment, can rapidly augment bone strength and provide an internal bond without compromising sternal perfusion. The adhesive has osteoconductive properties and host osteoblasts can synthesize new bone within the porous network of the material without loss of structural support, fibrosis, or inflammation. These features have previously been confirmed in a single-photon emission computed tomography (SPECT) bone scan study [11].

The objective of our study was to examine the possible benefits of the use of a special “bone cement enhanced” sternum closure technique in patients undergoing conventional cardiac surgery. In addition, the effect of risk factors on the occurrence of sternal dehiscence were assessed.

Osteoporosis, a systemic disease commonly occurring in elderly patients, is a major risk factor for sternal dehiscence and wound infection after median sternotomy due to fragility of the sternum. Spreader blades can damage sternal edges during insertion and spreading. Similarly, steel sutures can easily cut the sternum after they are tied, and spreader and steel sutures can cause transverse fragmentation or longitudinal separation of the sternum [12,13]. Although the elderly comprised majority of our study population, no cases of dehiscence were observed. Furthermore, three patients between 80 and 89 years of age did not experience any sternal complications despite undergoing vertebral surgery during the early postoperative period.

Obesity is also, a major risk factor for sternal dehiscence with or without infection after any type of cardiac operation. Of our study group, 64% were overweight and 26% were obese. Among these subjects, clinical examination showed minimal instability in two patients that correlated with the gaps on CT images. Sternal gaps are a relatively common finding even in asymptomatic patients and are not necessarily indicative of sternal dehiscence. Minor gaps (1–3 mm) do not correlate with any clinical sternum instability [14].

COPD represents another risk factor for sternal dehiscence. Physical forces generated by coughing can cause dehiscence or separation of the sternal halves. A successful sternal closure must be able to survive cyclic loading from repetitive physiological forces. Increased risk is also associated with the fact that sternotomy results in significant postoperative atelectasis and reduces pulmonary functions. In our study, 40% of the patients had COPD and all achieved sternal union as demonstrated by CT.

The reported incidence of dehiscence after coronary artery bypass grafting varies between 1.2 and 2.5%. Use of the internal mammary artery (IMA) for coronary revascularization may cause further increase in this risk. After removal of the main source of sternal blood supply and disruption of the cross-anastomoses between the periosteal plexus by median sternotomy, the collateral flow from intercostal and segmental branches cannot immediately substitute for the missing MA [15]. When both IMAs are used, the incidence of sternal and mediastinal sepsis may be as high as 8% [16]. In our study, 32 patients underwent coronary artery bypass surgery and both IMAs were utilized in 5 patients. However, no cases of dehiscence or infection were found.

Postoperative pain correlates with stability and alignment of the sternal closure. Early postoperative pain can result in poor mobility and shallow breathing that may lead to complications, including deep vein thrombosis, pulmonary embolus and pneumonia. It is also worth remembering that the use of strong narcotics in the elderly patients can have serious side effects such as hallucinations, confusion and disabling falls [17]. In this regard, kryptonite bone cement was previously shown to be associated with significantly less pain, less requirement for analgesia, and a faster return to baseline pulmonary functions in a total of 34 patients [18]. Similarly, most of our patients did not experience severe postoperative pain with kryptonite bone cement, including those with arthritic conditions.

Patient recovery and return to normal activities after cardiac operations is often delayed by a 6 to 8 week period required for osteosynthesis (solid bony union). In most centers, the recommended duration for sternal precaution is 6 to 8 weeks. Due to physical changes occurring through ageing and presence of disabilities, this period may be even longer in the elderly jeopardizing daily functions. In a previous study, patients with adhesive-enhanced closure have been shown to have accelerated recovery of health-related quality of life and a reduction in overall physical disability in the postoperative period compared with control patients [18]. Similarly, reduced postoperative pain, improved respiration, increased mobility and accelerated recovery have been observed in our patients, resulting in good quality of life after conventional cardiac surgery. Particularly, accelerated recovery in the elderly was similar to that of younger patients. The quality of life was better in male patients than in females, probably due to gender differences in postoperative psychology.

### Limitations

Our sample size was small and there were no control patients. In addition, thorax CT scans were not performed at uniform time intervals.

## Conclusions

Kryptonite bone cement as an enhancement to conventional wire cerclage for sternal closure is a safe, simple and quick procedure requiring no special technical skills and is associated with accelerated sternal healing, reduced postoperative pain, and improved quality of life. Longer-term studies with larger sample size may be warranted to better define potential risks and benefits of this innovative technique.

## Abbreviations

MCT: Multislice computed tomography; BMI: Body mass index; SF-36: Short form- 36; PCS: Physical component of summary; MCS: Mental component of summary; COPD: Chronic obstructive pulmonary disease; SPECT: Single- photon emission computed tomography; IMA: Internal mammary artery.

## Competing interest

The authors declare that they have no competing interests.

## Authors’ contributions

ZB participated in sequence alignment and drafted the manuscript. YD and MB collected patient data. OLU contributed to the study by radiological analysis. BC, ES and BA participated in the design and coordination. All authors read and approved the final manuscript.
